# Predicting Coupled Herbs for the Treatment of Hypertension Complicated with Coronary Heart Disease in Real-World Data Based on a Complex Network and Machine Learning

**DOI:** 10.1155/2022/8285111

**Published:** 2022-01-22

**Authors:** Jia-Ming Huan, Yun-Lun Li, Xin Zhang, Jian-Liang Wei, Wei Peng, Yi-Min Wang, Xiao-Yi Su, Yi-Fei Wang, Wen-Ge Su

**Affiliations:** ^1^School of Traditional Chinese Medicine, Shandong University of Traditional Chinese Medicine, Jinan 250014, China; ^2^First School of Clinical Medicine, Shandong University of Traditional Chinese Medicine, Jinan 250014, China; ^3^The Affiliated Hospital of Shandong University of Traditional Chinese Medicine, Jinan 250014, China

## Abstract

Hypertension and coronary heart disease are the most common cardiovascular diseases, and traditional Chinese medicine is applied as an auxiliary treatment for common cardiovascular diseases. This study is based on 3 years of electronic medical record data from the Affiliated Hospital of Shandong University of Traditional Chinese Medicine. A complex network and machine learning algorithm were used to establish a screening model of coupled herbs for the treatment of hypertension complicated with coronary heart disease. A total of 5688 electronic medical records were collected to establish the prescription network and symptom database. The hierarchical network extraction algorithm was used to obtain core herbs. Biological features of herbs were collected from public databases. At the same time, five supervised machine learning models were established based on the biological features of the coupled herbs. Finally, the K-nearest neighbor model was established as a screening model with an AUROC of 91.0%. Seventy coupled herbs for adjuvant treatment of hypertension complicated with coronary heart disease were obtained. It was found that the coupled herbs achieved the purpose of adjuvant therapy mainly by interfering with cytokines and regulating inflammatory and metabolic pathways. These results show that this model can integrate the molecular biological characteristics of herbs, preliminarily screen combinations of herbs, and provide ideas for explaining the value in clinical applications.

## 1. Introduction

Every year, 10.4 million people die of complications of hypertension worldwide [1]. Organ damage caused by hypertension and cardiovascular disease (CVD) are currently the main causes of death [2]. Due to the abnormal increase in arterial pressure, the coronary artery is more likely to experience increased local tensile stress, causing endothelial injury, the accumulation of lipid particles, induction of inflammatory reactions, and acceleration of the growth of plaques [3]. More than 50% of hypertension sufferers have multiple cardiovascular risk factors, and 25%–30% of coronary heart disease (CHD) patients have hypertension [4–6].

Traditional Chinese medicine (TCM) is recommended as a complementary and alternative therapy in the treatment of hypertension and CHD in China. An existing systematic review shows that TCM herbs can improve the vascular endothelial function of patients with hypertension, inhibit inflammatory reactions, regulate blood lipids, and improve mood. Adjuvant TCM therapy more easily achieves the targeted blood pressure and improves the comfort of patients, protecting target organs and reducing cardiovascular events [7–9].

In the theory of TCM, herbs are combined to form a prescription according to the principle of “Jun-Chen-Zuo-Shi.” Among them, the herbs of “Jun” that play a core role are often applied as drug combinations, usually no more than three herbs for the core disease. On the other hand, there are obvious individual differences in concurrent diseases. TCM theory emphasizes that medication for individual patients should have suitable herb pairing rules. But it is difficult to reflect its curative effect by relying on traditional randomized controlled trials (RCTs). To fully reflect the disease characteristics of the individual and groups of patients, it is necessary to analyze them on the basis of real-world data (RWD) [10]. The traditional summary of drug compatibility is often based on the physician's long-term diagnosis and treatment experience, and the screening period takes too long to apply to rapidly changing disease spectrum. Through the algorithm analysis of RWD, we can quickly find the drug compatibility information hidden in the effective prescriptions, carry out bioinformatics analysis to achieve preliminary screening, and provide ideas for further experimental verification and clinical applications to accelerate and summarize the application rules of herbs.

Therefore, we developed a mining method based on RWD to explore effective coupled herbs for the treatment of hypertension complicated with CHD by combining their symptom information and target information. Existing network pharmacology and bioinformatics techniques have been widely used to discover the core targets of herbs, and the understanding of multiple targets with TCM therapy has become increasingly profound [11, 12]. However, most of the existing studies are based on the relationship between herbs, compounds, targets, and diseases. Previous studies [13] used the Dijkstra algorithm to integrate the symptom information emphasized by TCM into the herb information network. However, it is still unable to fully evaluate the closeness of herb combinations and complicated diseases, including the target similarity of different herbs and their contribution to the curative effect.

Supervised machine learning can aggregate a variety of herb feature information to generate a model, matching the input features of the herbs with the expected output to form a learning function and to complete the classifier after adjusting the parameters by cross-validation. Commonly used models include K-nearest neighbor (KNN), support vector machine (SVM), gradient boosting decision tree (GBDT), Bayesian network (BN), and logistic regression (LR). KNN is more sensitive to the local information in the feature space of the input herbs, while SVM and others reflect its global characteristics.

In this study, we established a prescription database and symptom database of patients with hypertension complicated with CHD. First, we used the hierarchical network extraction algorithm to extract the main herbs and symptoms from the database, collected biological information, established a biological network, including herbal compounds, targets, and related disease symptom information, and then used supervised machine learning models compared with the classical Apriori algorithm. The best model was used to evaluate the pertinence of each coupled herb in the treatment of hypertension complicated with CHD.

## 2. Materials and Methods

### 2.1. Data Preparation

In this study, 5688 electronic medical records (EMRs) of hypertension complicated with CHD collected from the Affiliated Hospital of Shandong University of Traditional Chinese Medicine (between July 1, 2014, and May 31, 2017) were used to extract and standardize the symptoms in the prescription and medical history of TCM [14], and the prescription database and symptom database were established. After that, we took the herbs in the prescription database as the node and the frequency of the two herbs as the weight. The hierarchical network extraction algorithm uses Liquorice software [15] to obtain the core herbs of the prescription network based on the degree coefficient prescription = 1.9.

#### 2.1.1. Identifying Compounds and Targets of Core Herbs

We identified compounds and targets of core herbs from online public databases: Traditional Chinese Medicine Systems Pharmacology Database and Analysis Platform (TCMSP) [16], SymMap [17], and The Encyclopedia of Traditional Chinese Medicine (ETCM) [18], and published biomedical literature in the PubMed and CNKI databases. The names of the compounds were merged after being unified by PubChem and UniProt. The target of the core herbs was imported into the STRING database [19], and a protein–protein interaction network (PPIN) with a confidence ≥0.9 of *Homo sapiens* was established.

#### 2.1.2. Identifying the TCM Symptoms of Core Herbs

The SymMap database contains TCM symptoms corresponding to herbs and their compounds, and the TCM symptoms of the core herbs can be obtained from the SymMap database and compared with the symptom database in EMRs to screen and determine the symptoms of hypertension complicated with CHD that can be effectively treated by each herb.

#### 2.1.3. Collecting the Related Genes of Hypertension and CHD

The expression data of hypertension and CHD were retrieved from MalaCards [20] and the NCBI GEO database. The GSE76845 dataset contains 5 hypertension patient samples and 5 healthy control samples. The GSE71226 dataset contains 3 coronary heart disease patient samples and 3 healthy control samples. Then, the differentially expressed genes with *Q* < 0.05 and adj. *P* < 0.05 were analyzed by the GEO2R tool.

### 2.2. Evaluating the Features of the Coupled Herbs

The coupling of herbs can increase the pertinence of disease treatment. In this study, the core herbs coupled with each other. The main clinical and biological features of the herbs were quantified to evaluate the action characteristics of the coupled herbs.

#### 2.2.1. Frequency Assessment of the Coupled Herbs

To evaluate the correlation of the two matched herbs in the prescription database, we established the frequency matrix of the herbs in the prescriptions. The Manhattan distance [21] between two herbs was calculated to evaluate their coupling characteristics in the prescription.

#### 2.2.2. Symptom Similarity Assessment

The Jaccard similarity coefficient is used to compare the similarity between the sample sets. We obtained the symptom set of hypertension complicated with CHD and the herbal regulation symptom sets by screening the information of the RWD symptom database and the SymMap database. The Jaccard similarity coefficient was used to compare the similarity between the sample sets. We obtained the TCM symptom information of compound-regulated hypertension complicated with CHD by screening the information of the EMRs symptom database and the SymMap database, which was used to calculate the Jaccard similarity coefficient to evaluate the closeness of the herb-related symptoms to the disease-related symptoms.

#### 2.2.3. Bioavailability Assessment

Oral bioavailability (OB) represents the percentage of oral doses reaching the systemic circulation, and high OB is usually a key indicator for identifying bioactive molecules as having therapeutic properties. In this study, the OB values of the herbal compounds obtained from the SymMap database were added to evaluate the bioavailability of the coupled herb.

#### 2.2.4. Herb Target Identification and Functional Enrichment Analysis

A full understanding of human biological function cannot be realized by individual genes, only by the ubiquitous interaction between different genes. Therefore, the random walk with restart (RWR) algorithm was used in PPIN to evaluate the connection degree of herbal targets to disease-related genes. The disease-related genes in PPIN were used as seed node sets, and the restart probability was 0.75 [22]. The stable probability of diffusion to each herbal target was obtained by the RWR operation, which was realized in the *PyRWR* package (version 1.0.0) in Python 3.7.5. The summation was used to evaluate the regulation of disease-related proteins of herbs.

The semantic comparison of gene ontology (GO) annotations provides a method to calculate the similarity between genes and genomes. To measure the similarity between herbal targets and disease-related genes, GO biological process semantic similarity (GoSim) was used to evaluate its effectiveness. We relied on the annotated data provided by *Bioconductor* and used the algorithm designed by Wang et al. [23], implemented in the *GOSemSim* package [24] (version 2.12.1) in R 3.6.3.

### 2.3. Machine Learning Model Training

**Figure 1:**
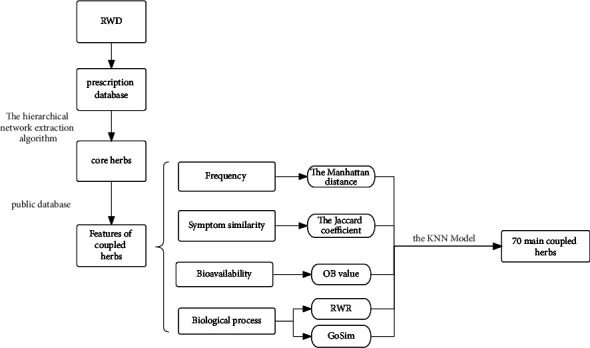
Overview of herb screening: first, we used the hierarchical extraction algorithm to analyze the core herbs from the prescription database. Second, we obtained the features of the coupled herbs from a public database. Finally, we input the features into the machine learning model to obtain 70 main coupled herbs.

We took the lift value of the coupled herbs calculated by the Apriori algorithm as the classification criterion and the evaluation value of the effectiveness of the coupled herbs as the input information for the machine learning tasks (Figure 1) and used the *kknn* (version 1.3.1), *e*1071 (version 1.7-8), *gbm* (version 2.1.8), and *klaR* (version 0.6-15) packages in R 3.6.3 to complete the model training of KNN, SVM, GBDT, BN, and LR. Ten cross-validations were used to evaluate the performance of the models. We, in particular, randomly divide the performance data of the coupled herbs into 10 subsets, selected one as the test set in turn, and repeated the other 9 training sets 10 times. According to the verification of a large amount of data [25], 10% can obtain the best error estimate, which can be used to prevent overfitting of the model, and all drug pair data can be used as training sets and test sets to effectively avoid data waste.

### 2.4. Analysis of the Mechanism of Coupled Herbs

We used the *clusterProfiler* package [26] (version 3.14.3) in R 3.6.3 to annotate genes with *org.Hs.eg.db* (version 3.10.0) and analyzed the related genes of the effective coupled herbs by KEGG enrichment analysis. Based on the hypergeometric distribution, *Q* < 0.05 was considered a significant enrichment pathway, and the same method was used for GO enrichment analysis, *Q* < 0.05 and adj. *P* < 0.05 as significant enrichment. Hierarchical clustering (HCT) was used to classify the herbs and pathways to distinguish the biological processes of intervention. At the same time, we introduced genes into *Metascape* [27] for multigene list meta-analysis, including functional proteomics and gene screening.

## 3. Results

### 3.1. The Core Herbs

A total of 5,689 electronic medical records data of Chinese medicines for the treatment of hypertension and CHD were collected to establish a database. Among them, the TCM prescription database contains 3697 prescriptions, which included 442 herbs used 85662 times, and each prescription contained, on average, 23.17 ± 10.38 herbs. A total of 234 disease-related symptoms were found in the symptoms database. Through the hierarchical extraction algorithm, we obtained 18 core herbs, including Atractylodis Macrocephalae Rhizoma, Citri Reticulatae Pericarpium, Glycyrrhizae Radix Et Rhizoma, Rhizoma Pinelliae, Poria, Radix Salviae Liguliobae, Codonopsis Radix, Rhizoma Coptidis, Radix Angelicae Sinensis, Astragali Radix, Radix Paeoniae Alba, Puerariae Lobatae Radix, Chuanxiong Rhizoma, Radix Notoginseng, Ophiopogonis Radix, Rehmanniae Radix, Schisandrae Chinensis Fructus, Ziziphi Spinosae Semen.

**Figure 2:**
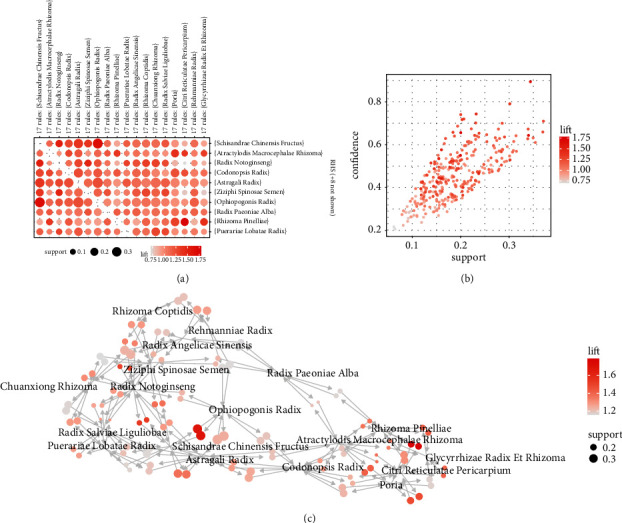
Results of apriori: (a) and (c) are the paired relationships between the core herbs obtained by the apriori algorithm. The size of the dot indicates the support between two herbs, and the darker the color of the dot, the larger the lift. (b) shows the coupling feature of all herbs obtained by the apriori algorithm.

After 18 herbs were coupled with each other, 77 coupled herbs (Figure 2) were selected as the main herb pairs through the Apriori algorithm, of which Schisandrae Chinensis Fructus, Codonopsis Radix, Astragali Radix, Radix Notoginseng, Chuanxiong Rhizoma, Ziziphi Spinosae Semen, Radix Angelicae Sinensis, Atractylodis Macrocephalae Rhizoma, Radix Paeoniae Alba, and Rhizoma Coptidis accounted for a high proportion (Table 1).

### 3.2. Genes Related to Hypertension Complicated with CHD

**Figure 3:**
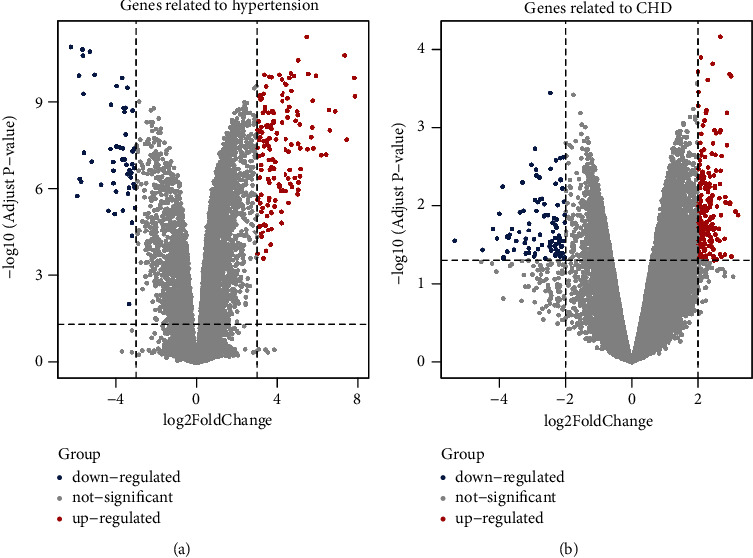
Genes related to hypertension (a) and CHD (b): a blue dot indicates a downregulated gene, and a red dot indicates an upregulated gene.

As shown in Figure 3, 211 hypertension-related genes were obtained from GSE76845. The main upregulated genes included RNF6, UBE2L5, MTX1, FTL, SLITRK2, OGG1, HMGA1, RHAG, and TRIM8. The main downregulated genes included SPAG17, ALPG, ENO1, ESYT2, DNAJB1, HFE, RAPSN, DLGAP5, and OST4. Then, 246 CHD-related genes were obtained from GSE71226. The main upregulated genes included TM2D1, SRSF11, SLC33A1, NFATC2IP, GPRIN3, FRG1JP, DROSHA, and CHD2. The main downregulated genes included YBX3, TMCC3, OAZ2, MME, KRTTN1, IL1R2, GYPA, and HYPA. MalaCards obtained 33 hypertension-related genes and 20 CHD-related genes.

### 3.3. Performance of the Models

**Figure 4:**
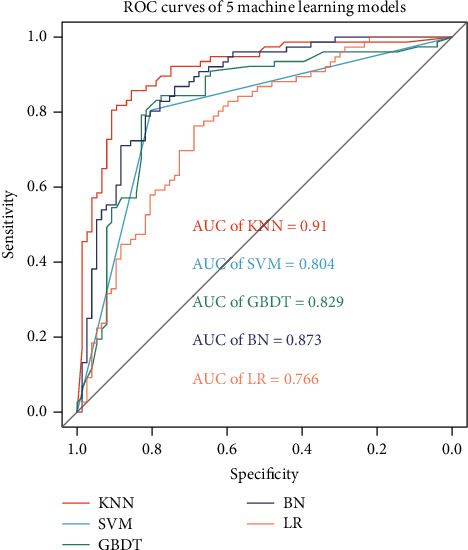
ROC curves of 5 machine learning models.

In this study, 5 machine learning models, KNN, SVM, GBDT, BN, and LR, were used to analyze the effectiveness feature data of the coupled herbs and establish the coupled herbs classification. As shown in Figure 4, the area under the receiver operating characteristic (ROC) curve (AUROC) was used to evaluate the performance of the models. We found that the 5 models had good classification performance. Among them, the KNN effect was the best, with an AUROC of 91.0%. BN, GBDT, and SVM were all over 80%, 87.3%, 82.9%, and 80.4%, respectively. The GBDT was 76.6%.

### 3.4. Prediction of the Coupled Herbs

The performance of the KNN model was the best when the parameter *k* was 9. According to its best cutoff point value, we obtained a herb combination relationship network between the paired drugs containing 18 herbs. There were 70 main coupled herbs. The top 15 effective coupled herbs screened by the KNN model are shown in Table 2. Combining the information of Tables 1 and 2, we found that Schisandrae Chinensis Fructus, Codonopsis Radix, Radix Notoginseng, Atractylodis Macrocephalae Rhizoma, Ziziphi Spinosae Semen, and Radix Paeoniae Alba were the 6 herbs most related to the other drugs. The percentages of Radix Notoginseng, Atractylodis Macrocephalae Rhizoma, Ziziphi Spinosae Semen, and Radix Paeoniae Alba increased compared to the result of Apriori, while the percentages of Astragali Radix, Chuanxiong Rhizoma, and Radix Angelicae Sinensis decreased. At the same time, there were Citri Reticulatae Pericarpium-Rhizoma Pinelliae, Citri Reticulatae Pericarpium-Poria, Schisandrae Chinensis Fructus-Ophiopogonis Radix, and Radix Notoginseng-Rhizoma Coptidis which are individually strongly associated drugs.

### 3.5. Validation of the Models' Effect

To verify the effect of the KNN model, we used the data diagnosed as hypertensive nephropathy (HN) in the standardized electronic medical record data from the Affiliated Hospital of Shandong University of Traditional Chinese Medicine. GEO2R was used to analyze GSE99325 to obtain HN-related genes. The final AUROC obtained using the KNN model was 94.2% with parameter *k* being 4. The effective therapeutic coupled herbs obtained include Radix Salviae Liguliobae-Astragali Radix, Radix Achyranthis Bidentatae-Radix Angelicae Sinensis, Astragali Radix-Radix Angelicae Sinensis, and Ophiopogonis Radix-Poria. According to recent HN-related studies [28–32], all of the coupled herbs screened in the KNN model have therapeutic effects on HN.

### 3.6. Therapeutic Mechanism of the Coupled Herbs

The targets of the core herbs were analyzed by GO and KEGG enrichment analysis, and the pathways in which the number of enriched genes for each herb was greater than the quartile of related genes of the herb were retained.

**Figure 5:**
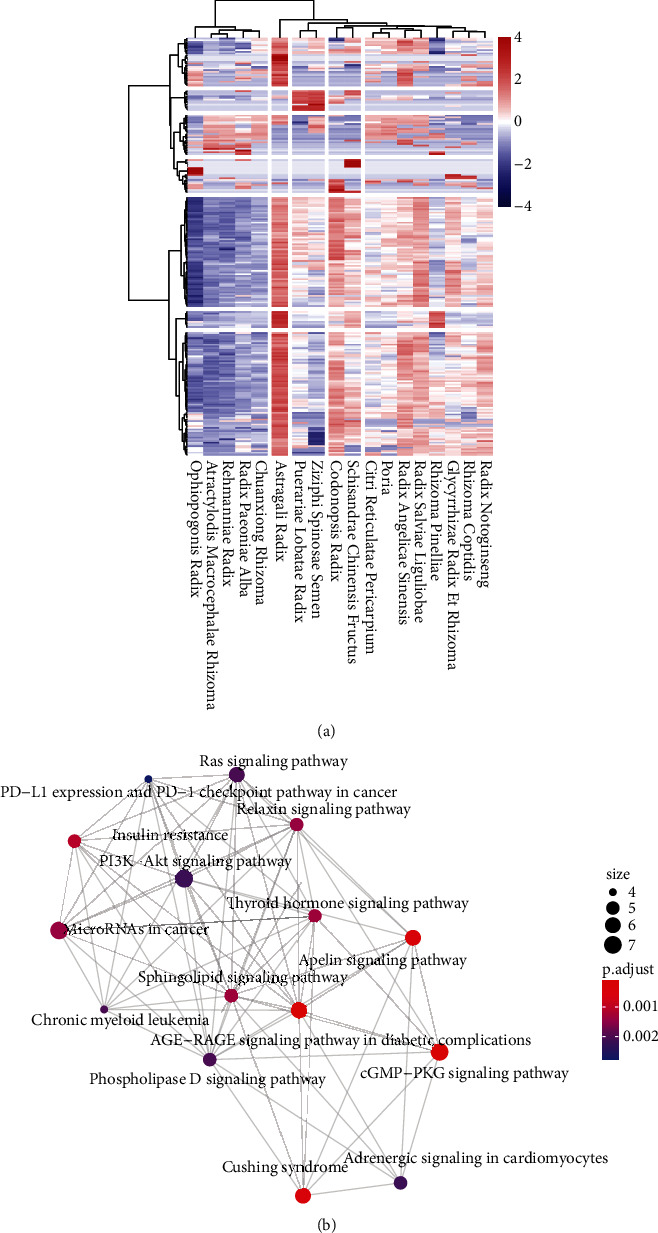
The results of KEGG pathway enrichment: heatmap and hierarchical clustering result of the KEGG signaling pathways (a) and the KEGG pathway network (b).

A total of 215 KEGG signaling pathways are obtained. We established an 18 × 215-dimensional feather profile of the core pathways. HCT divided the KEGG signaling pathways into 7 parts. As shown in Figure 5, based on the KEGG signaling pathways, 18 herbs were divided into 5 parts, each with similar enrichment results. The reserved coupled herbs screened by the KNN model were 59 in different clusters and 10 in the same cluster. Among the excluded coupled herbs, there were 59 in different clusters and 25 in the same cluster. In the reserved coupled herbs, herbs in different clusters were more abundant (*P*=0.04). In addition, the number of pathways in which the number of enriched genes was greater than the average number of herbs coupled with herbs in different clusters was larger (*P* < 0.01), indicating that the biological processes of the pairing of herbs in different clusters were more extensive.

For example, Atractylodis Macrocephalae Rhizoma, Ophiopogonis Radix, Radix Paeoniae Alba, Chuanxiong Rhizoma, and Ziziphi Spinosae Semen in K1 regions have fewer enriched genes in the inflammatory pathways, such as the HIF-1 signaling pathway, TNF signaling pathway, NOD-like receptor signaling pathway, NF-kappa B signaling pathway, and 31 coupled herbs related to them; 16 were combined with herbs in the K5 region to supplement the regulation of the inflammatory pathway. At the same time, compared with the K5 region, the K1 region had fewer enriched genes in glucose and lipid metabolism, such as insulin resistance, insulin secretion, cholesterol metabolism, and the regulation of lipolysis in adipocytes, so it was necessary to combine the K5 region herbs. A total of 37.5% of the coupled herbs contained herbs in K2 and K4, and the herbs in K4 were mainly enriched in leukocyte transendothelial migration, the calcium signaling pathway, platelet activation, and other pathways related to the formation of coronary artery plaques.

**Figure 6:**
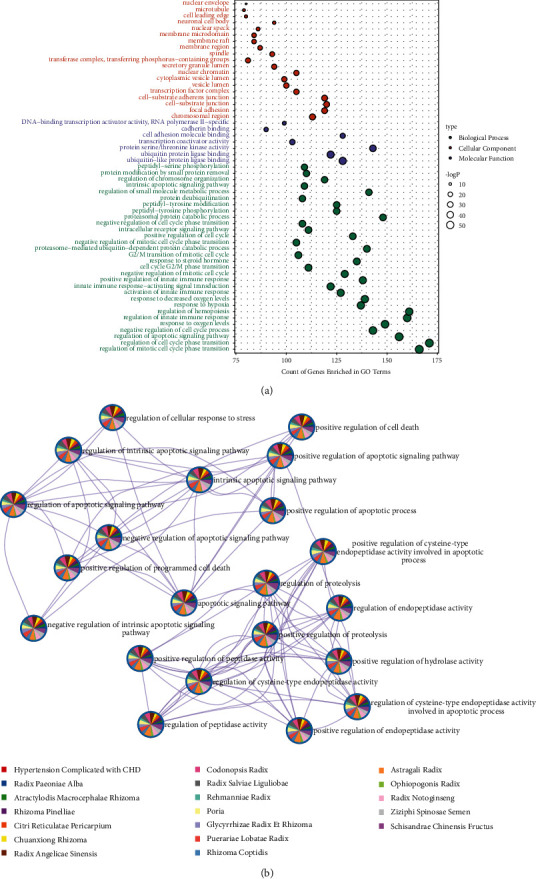
The results of the GO term enrichment: (a) is the enrichment result of biological process, cellular component and molecular function. (b) is a network of enriched GO terms represented as pie charts, where pie pieces are color-coded based on the identities of the genes in the herbs and hypertension complicated with CHD. The thicker the line, the more common the targets of the nodes and the closer their interaction.

GO enrichment found that the number of enriched genes in the biological process (BP) category of the KNN-coupled herbs was different from that of the deleted coupled herbs (*P*=0.04), and the molecular function (MF) and cellular component (CC) categories were not different. From the results of GO enrichment in Figure 6(a), the regulatory effects of the core herbs were mainly cytokines, chemokines, growth factors, and the regulation of cell metabolism. As shown in Figure 6(b), the regulatory proteins of the core herbs and the genes related to hypertension complicated with CHD can be coenriched on the main nodes of the GO term network, suggesting that the paired core herbs can cooperate and complement each other, resulting in the regulation of hypertension complicated with CHD.

## 4. Discussion

The prescription rule of “Jun-Chen-Zuo-Shi” means that different combinations of herbs in the prescription need to be effective against multiple symptoms of clinical diseases. At the same time, the combined use of herbs is in keeping with the TCM theory which emphasizes that the core herbs enhance the efficacy of specific disease symptoms. Herbs are rich in components and match the network regulation mechanism of the disease. Experiments verification for the total mechanism is tedious and expensive. In recent years, with advances in bioinformatics research and the advent of the concept of multitarget drugs, information on compounds and targets has been extracted and screened in large herb databases and biological databases. Evaluating and predicting the efficacy of herb compound prescriptions and deducing their action mechanism has become a widely used research model [13, 15, 33].

The existing herbal pairing research is based on a single commonly used prescription, establishes a drug-gene-disease network and explores the correlation characteristics between nodes, analyzes the interaction law of herbal combinations after prescription decomposition, and discusses the rationality of its curative effect [34–36]. However, in clinical practice, such prescriptions are often used as “Jun” or “Chen” in patients' prescriptions to regulate major disease symptoms, which seriously limits the summary of the findings on the treatment of concurrent diseases and symptoms. Therefore, this study used 5688 EMRs as the data source, extracted the patients' TCM symptoms, and used the hierarchical network extraction algorithm to extract common drugs for the treatment of hypertension complicated with CHD in the prescription network, which is not limited to a single doctor or genre. Treatment experience can be widely collected and summarized, but it also increases the heterogeneity of the dataset. To screen out truly effective paired drugs, we established a prediction model for screening the effectiveness of herbs, which integrates different types of information, including the bioavailability of compounds, the degree of association between herbs and the clinical symptoms and the biological functions.

According to previous research [33], the effective coupled herbs in the complete prescriptions of TCM include drugs with different pharmacological directions and application frequencies. Therefore, this study quantifies the effectiveness of coupled herbs on the basis of PPIN. First, we incorporate TCM symptom information into herbal PPIN but, at the same time, it also brings more noise and biological dimensions. By using RWR to amplify the connection information between nodes, the connection probability between herbal targets and disease symptom-related proteins was evaluated, and their closeness was quantified. At the same time, we used Manhattan distance to evaluate the correlation between the two herbs in the clinical application, the Jaccard coefficient to evaluate the similarity between paired herbal symptoms and the disease symptom database, and the OB to determine the bioavailability of the herbs. After calculating the effectiveness of five classical classification machine learning models, the best KNN is selected as the classification model with an AUROC of 91.0%, and when the HN data are used to evaluate the effectiveness of the model, the AUROC is 94.2%. Therefore, according to the best cutoff point of the KNN model, we finally identified 70 effective coupled herbs.

Hypertension and CHD are common cardiovascular diseases with overlapping risk factors. After an increase in blood pressure, the renin–angiotensin–aldosterone system (RAAS) is overactivated, which induces an inflammatory reaction of target organs such as blood vessels, myocardium, and kidney, resulting in the upregulation of inflammatory cytokines. The coronary artery induces vascular endothelial injury, promotes lipids to enter the intima, causes platelet aggregation, and accelerates the growth of plaques [3, 37–39]. To further analyze the therapeutic pathways of coupled herbs, we carried out GO and KEGG enrichment analysis on each herbal target. Finally, we found that coupled herbs can cooperate with each other, directly or indirectly acting on the inflammatory pathway, further assist the body in controlling risk factors by regulating blood lipids, proteins, and carbohydrate metabolism, regulate the expression of various cytokines at the cellular level, and regulate their target proteins to inhibit the inflammatory response and interstitial fibrosis of target organs caused by hypertension.

The analysis results of GO and KEGG enrichment showed that the herbal combinations with synergistic effects could be correctly identified by a machine learning model with the quantitative indicators of herbal effectiveness used in this study, but this study also contains many limitations. First, the herbal-related compounds and target information come from public database. Although we used a variety of models to compare and verify to find the best model, the data deviation of the database may have a potential impact on the research results. Second, although the TCM symptom information of patients has been included in the evaluation system, it fails to make full use of the clinical information of individual differences in EMRS. Third, the sample size for model training is still limited, and information on the interaction between compounds has not been added, so future research needs to improve the performance of the model to design a more effective adjuvant therapy for TCM.

## 5. Conclusion

This study was based on data from 5688 EMRs of hypertension complicated with CHD in the Affiliated Hospital of Shandong University of Traditional Chinese Medicine from 2014 to 2017, and a patient symptom dataset and prescription dataset were established. Eighteen commonly used herbs were obtained, and their biological network was established. Using the interaction information between nodes in the network, we established quantitative data on the effectiveness of coupled herbs. An effective coupled herb screening algorithm based on a machine learning model was proposed, and a total of 70 coupled herbs were obtained. Based on the analysis of various herbs at the pathway level, they can play a multilevel biological regulatory role in controlling the inflammatory response and regulating energy metabolism for hypertension complicated with CHD and its TCM symptoms. Combined with complex networks and machine learning, this study explored the potential law of herbs in the treatment of hypertension complicated with CHD and predicted the curative effect of coupled herbs, providing a direction for summarizing the TCM EMRs and explaining the TCM rules.

## Figures and Tables

**Table 1:** Regional division of the core herbs.

Core herbs	Proportion in the main coupled herbs		Regional division
	According to apriori (%)	According to the KNN model (%)	
Radix paeoniae alba	5.84	6.52	K1
Atractylodis macrocephalae rhizoma	5.84	7.97	K1
Chuanxiong rhizoma	6.49	5.80	K1
Rehmanniae radix	4.55	1.45	K1
Ophiopogonis radix	4.55	5.07	K1
Astragali radix	6.49	5.80	K2
Puerariae lobatae radix	5.19	5.07	K3
Ziziphi spinosae semen	6.49	7.25	K3
Codonopsis radix	7.14	7.97	K4
Schisandrae chinensis fructus	8.44	7.97	K4
Rhizoma pinelliae	5.19	5.07	K5
Citri reticulatae pericarpium	3.90	4.35	K5
Radix angelicae sinensis	6.49	5.80	K5
Radix salviae liguliobae	4.55	4.35	K5
Poria	3.25	2.90	K5
Glycyrrhizae radix et rhizoma	3.25	2.90	K5
Rhizoma coptidis	5.84	5.80	K5
Radix notoginseng	6.49	7.97	K5

**Table 2:** The top 15 effective coupled herbs screened by the KNN model.

Coupled herbs	
Astragali radix	Ophiopogonis radix
Ophiopogonis radix	Schisandrae chinensis fructus
Atractylodis macrocephalae rhizoma	Citri reticulatae pericarpium
Citri reticulatae pericarpium	Poria
Rhizoma pinelliae	Citri reticulatae pericarpium
Radix notoginseng	Ziziphi spinosae semen
Chuanxiong rhizoma	Radix angelicae sinensis
Codonopsis radix	Schisandrae chinensis fructus
Ziziphi spinosae semen	Schisandrae chinensis fructus
Radix paeoniae alba	Atractylodis macrocephalae rhizoma
Rhizoma pinelliae	Poria
Puerariae lobatae radix	Radix notoginseng
Atractylodis macrocephalae rhizoma	Poria
Radix paeoniae alba	Codonopsis radix
Radix notoginseng	Schisandrae chinensis fructus

## Data Availability

The TCM prescription data used to support the findings of this study are available from the corresponding author upon request. The data generated by the analysis process can be found in the article databank.

## Abbreviations

CVD: Cardiovascular disease
CHD: Coronary heart disease
TCM: Traditional Chinese medicine
RCT: Randomized controlled trial
RWD: Real-world data
KNN: K-nearest neighbor
SVM: Support vector machine
GBDT: Gradient boosting decision tree
BN: Bayesian network
LR: Logistic regression
EMR: Electronic medical record
TCMSP: Traditional Chinese Medicine Systems Pharmacology Database and Analysis Platform
ETCM: The Encyclopedia of Traditional Chinese Medicine
PPIN: The protein–protein interaction network
RWR: Random walk with restart
GO: Gene ontology
GoSim: Gene ontology biological process semantic similarity
OB: Oral bioavailability
HCT: Hierarchical clustering
Baizhu: Atractylodis Macrocephalae Rhizoma
Chenpi: Citri Reticulatae Pericarpium
Gancao: Glycyrrhizae Radix Et Rhizoma
Banxia: Rhizoma Pinelliae
Fuling: Poria
Danshen: Radix Salviae Liguliobae
Dangshen: Codonopsis Radix
Huanglian: Rhizoma Coptidis
Danggui: Radix Angelicae Sinensis
Huangqi: Astragali Radix
Baishao: Radix Paeoniae Alba
Gegen: Puerariae Lobatae Radix
Chuanxiong: Chuanxiong Rhizoma
Sanqi: Radix Notoginseng
Maidong: Ophiopogonis Radix
Dihuang: Rehmanniae Radix
Wuweizi: Schisandrae Chinensis Fructus
Suanzaoren: Ziziphi Spinosae Semen
ROC: Receiver operating characteristic
AUROC: Area under the receiver operating characteristic curve
HN: Hypertensive nephropathy
Niuxi: Radix Achyranthis Bidentatae
Maidong: Ophiopogonis Radix
RAAS: Renin–angiotensin–aldosterone system.

## Supplementary Materials

Supplementary Materials 1. Apriori: rules of the core herbs obtained for the Apriori algorithm. 2. Features of coupled herbs: The value of Jaccard similarity coefficient, OB, RWR, GoSim, Manhattan distance, and KNN model output of coupled herbs. 3. The main coupled herbs: Seventy coupled herbs for adjuvant treatment of hypertension complicated with coronary heart disease screened by the KNN model. 4. TCM herbs information: the Chinese name, Pinyin name, Latin name, and English name of herbs in this study.
